# Explaining Income-Related Inequalities in Dietary Knowledge: Evidence from the China Health and Nutrition Survey

**DOI:** 10.3390/ijerph17020532

**Published:** 2020-01-15

**Authors:** Yongjian Xu, Siyu Zhu, Tao Zhang, Duolao Wang, Junteng Hu, Jianmin Gao, Zhongliang Zhou

**Affiliations:** 1 School of Public Policy and Administration, Xi’an Jiaotong University, Xi’an 710049, China; wgsxyj@xjtu.edu.cn (Y.X.); zhusiyu1996@stu.xjtu.edu.cn (S.Z.); juntenghu@xjtu.edu.cn (J.H.); gaojm@xjtu.edu.cn (J.G.); zzliang1981@xjtu.edu.cn (Z.Z.); 2 Department of Health Management, School of Medicine and Health Management, Tongji Medical College, Huazhong University of Science and Technology, Wuhan 430074, China; 3 Medical College, Hangzhou Normal University, Hangzhou 311121, China; 4 Department of Clinical Sciences, Liverpool School of Tropical Medicine, Liverpool L3 5QA, UK; Duolao.Wang@lstmed.ac.uk

**Keywords:** dietary knowledge, income-related inequality, concentration index, decomposition analysis

## Abstract

Lack of adequate dietary knowledge may result in poor health conditions. This study aims to measure income-related inequality in dietary knowledge, and to explain the sources of the inequality. Data were from the China Health and Nutrition Survey (CHNS) conducted in 2015. A summary of the dietary knowledge score and dietary guideline awareness was used to measure the dietary knowledge of respondents. The concentration index was employed as a measure of socioeconomic inequality and was decomposed into its determining factors. The study found that the proportion of respondents who correctly answered questions on dietary knowledge was significantly low for some questions. Compared to rural residents, urban residents had a higher proportion of correctly answered dietary knowledge questions. In addition, there are pro-rich inequalities in dietary knowledge. This observed inequality is determined not only by individual factors but also high-level area factors. Our study recommends that future dietary education programs could take different strategies for individuals with different educational levels and focus more on disadvantaged people. It would be beneficial to consider local dietary habits in developing education materials.

## 1. Introduction

Overweight and obesity are a worldwide public concern. The World Health Organization (WHO) estimated that 39% of women and 39% of men aged 18 and over were overweight in 2016 [1]. Previous evidence has shown that an unhealthy diet was admittedly one of the critical contributors to obesity. Previous systematic reviews which focused on the relationship between the nutrition knowledge and dietary intake showed that, as an integral component of health literacy, low nutrition knowledge has a significant association with the poor eating habits and bad health conditions [2]. Health literacy includes the level of knowledge, personal skills and confidence to take action to improve personal and community health by changing personal lifestyles and living conditions [3].

As result of adopting open up policy and reformation in recent decades, China has experienced expansive economic growth. The dietary problems remarkably transited from malnutrition due to food scarcity in whole country to the excessive and unbalanced nutrition due to dietary structure and poor dietary habit. Unfortunately, the rapid growth in the economy has not provided improvement of diet quality to Chinese people. With the increase in income, people gradually converted from consuming high-carbohydrate and high-fiber foods to consuming high-fat, high-energy-density foods [4]. Unhealthy food also plays an important role in the development of the high prevalence of overweight and obesity. It is estimated by the National Health Commission of China that the prevalence of overweight and obesity jumped from 22.85% to 30.15% and 7.1% to 11.9% from 2002 to 2015, respectively [5]. People who are overweight or obese show potentially unhealthy changes in metabolism, and thus have a high risk of suffering from various chronic diseases. This may impose a heavy burden on the social security system of China [5].

Apart from the higher prevalence of unhealthy diet intake, it was noted that the unhealthy diet is distributed unequally among the different socioeconomic groups. The unhealthy diet is disproportionately concentrated on the poor and low-income individuals [6,7]. For many people, the direct culprit has long been the traditional Chinese diet. Low-income people are more likely to choose cheaper, high-calorie, and less nutritious food as their staple food resulted from limited nutritional food options. However, the more fundamental differences between the poor and the rich may be indirectly originated from their economic status, especially in education and dietary knowledge, which shape residents’ eating habits and in turn impact residents’ health [6,8]. It is believed that rich dietary knowledge leads to long-term changes in attitude about diet, influencing food choices and dietary quality, and consequently leading to better health conditions in the public.

In response to the increase in overweight and obesity in the population in the 1990s, the Chinese Government has implemented health education programs for rural and urban citizens to reduce the harmful health effects of an unhealthy diet. These programs aimed to improve people’s dietary knowledge, develop better eating habits and maintain healthy lifestyles. Accordingly, the Chinese Academy of Preventive Medicine and the Chinese Nutrition Society developed Dietary Guidelines, which was then published to assist health professionals and policy makers in guiding Chinese people to make healthy food and beverage choices. The guidelines serve as the science-based foundation for vital nutrition policies and programs across China. The Dietary Guidelines are updated every 8 to 10 years, and the latest version of the Dietary Guidelines for Chinese Residents was published in 2016.

Several studies conducted previously described the distribution of nutrition knowledge among specific groups and assessed the association between income and nutrition knowledge [9]. These studies consistently found that there was a disparity in nutrition knowledge between different income groups, resulting in lower income people having the lowest nutrition knowledge [10]. Although both dietary knowledge and nutrition knowledge emphasize the importance of healthy eating, nutrition knowledge is more concentrated on nutrients inside the food and its association with disease, whereas dietary knowledge focuses more on the importance of a balanced diet and physical exercise [5]. In a systematic review, despite the availability of a previous study focusing on nutrition knowledge, to date, there are very limited studies focusing on dietary knowledge [11,12]. These limited existing studies on dietary knowledge mainly focused on assessing the effect of health education intervention measures. These studies consistently showed that these measures could improve the level of residents’ dietary knowledge. This study explored the determinants of dietary knowledge, which was rarely investigated in the previous studies [13,14,15]. In addition, no study has been conducted to measure the magnitude of income-related inequality in dietary knowledge. Furthermore, studies on finding the socioeconomic aspect that causes this disparity between the advantaged and disadvantaged people are rarely reported in China. This paper, therefore, aims to fill in the gaps in the existing literature and provides more information by estimating the contribution of socioeconomic factors on inequality in dietary knowledge. The specific questions are as follows: (1) What is the distribution of dietary knowledge scores and what is the level of awareness of dietary guidelines among the Chinese? (2) Is there inequality in the dietary knowledge score and dietary guideline awareness between the poor and the rich? (3) What is the percentage of the determinant’s contribution towards the total inequality? The hypothesis is that there is income-related inequality in dietary knowledge, with the better off having more dietary related knowledge in China, and many determinants, such as income, education level, and geographic location, are contributors to this income-related inequality. This study will contribute to current studies by providing readers with a preliminarily understanding of the inequality in dietary knowledge. This study will support policy makers and public health workers with evidence-based information to address inequality as well as in developing health policy and public intervention measures.

## 2. Methods

### 2.1. Study Design

This is a cross-sectional study designed to measure the degree of inequality in dietary knowledge and decompose this inequality into its determinants.

### 2.2. Data

This study used data from the China Health and Nutrition Survey (CHNS). The CHNS is an ongoing international collaborative project between the Carolina Population Center at the University of North Carolina at Chapel Hill and the National Institute of Nutrition and Food Safety at the Chinese Center for Disease Control and Prevention. The CHNS collects the nutritional and health status, as well as the demographic and socioeconomic data of Chinese population [16]. The CHNS was designed to examine the effects of health policies and programs on the health and nutritional status of the Chinese population. The first round of the CHNS was conducted in 1989, and its successive rounds of the survey were conducted every 2−4 years. The latest survey, which included 20,914 individuals in 7319 households, was conducted in 2015. A multi-stage random cluster process was used to draw samples of approximately 7200 households, with over 30,000 individuals in 15 provinces and municipal cities. The provinces and cities vary substantially in geography, economic development, public resources, and health indicators. In each survey, a household interview, a health examination for children and adults, a nutrition survey, and a community survey were conducted simultaneously. The health examinations and household surveys were conducted in the participant’s home, while the community survey was collected by interviewing the neighborhood officials. The CHNS raw data are accessible through its official website (cpc.unc.edu/projects/china). Detailed information on survey contents and quality control procedures can also be found in the official website. This study focused on data of adult individuals from the latest round of the survey. Inclusion criteria were: (1) adults aged 18 years and over; (2) adults who can answer the questions independently. Exclusion criteria were: (1) households with missing data on health consumption and overall consumption expenditure; (2) respondents with missing data on studied variables. After data cleaning, 12,259 respondents in total were included in this study.

### 2.3. Variables

#### 2.3.1. The Dietary Knowledge Score

In the 2015 CHNS survey, 16 questions were preselected to assess the dietary knowledge of respondents. In each question, respondents were asked to choose “strongly disagree”, “disagree”, “neutral”, “agree” and “strongly agree”. For easier comparison with future studies, only 12 dietary knowledge items were selected in this study. In regression analysis, a summary dietary knowledge score was constructed by summing up the score of twelve questions. For each answer, we have constructed a score ranging from 0 to 2, with 2 for strongly agree, 1 for agree, and 0 for other answers in positive questions, and 2 for strongly disagree, 1 for disagree, and 0 for other answers in negative questions. The maximum dietary knowledge score is 24. A higher score indicates better knowledge.

#### 2.3.2. Dietary Guideline Awareness

Dietary guideline awareness is a binary variable, indicating whether the respondents know about the Dietary Guidelines for Chinese Residents, which is known as The Chinese Pagoda. The Dietary Guidelines for Chinese Residents is published by the National Health Commission of China. The Dietary Guidelines provides evidence-based food and beverage recommendations for Chinese aged 2 and over. The purpose of these recommendations is to promote population health, prevent the population from developing non-communicable diseases, help people to achieve a healthy weight, etc.

#### 2.3.3. Independent Variables

A great number of variables are available in the CHNS. Referring to previous nutrition studies, some demographic, socioeconomic, residential area and health education intervention variables were associated with nutritional knowledge [9,10,17,18]. Since this study was designed to explore the contribution of socioeconomic factors on dietary knowledge inequality, we constructed a hypothesis that all the socioeconomic variables and potential control variables in the CHNS are all associated with dietary knowledge. After removing redundant variables, four types of independent variables were included in this study to empirically examine the relationship between these variables and the dietary knowledge score/dietary guideline awareness. Demographic characteristics considered in this study were sex and age. Socioeconomic characteristics considered in this study were marital status (unmarried, married, divorced or widowed), educational attainment (illiterate, elementary, middle school, high school, and university), working status, basic medical insurance, and economic status (grouped equally into the poorest, poorer, middle, richer, and the richest quintiles according to the per capita net household expenditure). A set of location variables was included to capture the potential regional heterogeneity. These variables were geographic location (Eastern China, Central China, and Western China), residential area (urban or rural), and birthplace (North China, Northeast, East China, Central China, South China, and Western China). Lastly, the urbanization index, a comprehensive composite variable to measure the degree of urbanization on 12 aspects of urbanization in communities, was also included. The aspects of the urbanization index are population density, economic activity, traditional markets, modern markets, transportation, health infrastructure, sanitation, communication, social services, diversity and housing. Detailed information on this variable is available on the official CHNS website [19,20,21].

### 2.4. Statistical Analysis

Categorical variables are described as proportions (number and percentage), continuous variables are described as means and standard deviation (SD). The chi-square test was performed to compare the difference in dietary guideline awareness among different groups. One-way analysis of variance (ANOVA) and the t-test were used to assess the difference in the dietary knowledge score among groups. The post-hoc multiple comparison procedure was further employed to compare between subgroups.

The concentration index and the related concentration curve have become the most frequently used measures to quantify income-related inequalities in the health domain [22,23]. The concentration curve is displayed graphically, with the cumulative percentage of the population in income on the horizontal axis and the cumulative percentage of the health sector variable on vertical axis (for this study, the health sector variables are mentioned in the variables section). If the health sector variable has higher (lower) values among poorer people, the concentration curve will lie above (below) the line of equality. In the case that the health sector variables are distributed equally among all income groups, the concentration index would coincide with the 45-degree line [24,25]. The concentration index was employed to measure inequality in the health sector variables over the distribution of income. The concentration index is defined as twice the area between the concentration curve and the line of equality (the 45-degree line) [26]. The concentration index value is bound between −1 and +1. The negative concentration index indicated that the health sector variables were disproportionately concentrated among the poor, and vice versa [27]. The concentration index formula is as follows:

$$c=\frac{2}{\mu }\mathrm{cov}(y_{i},R_{i})$$

where *c* is the concentration index, $y_{i}$ is the health sector variable of individual *i*, $R_{i}$ is the fractional ranks of income, and μ is the mean of $y_{i}$.

The health concentration index can be decomposed into the contributions of each determinant to observed income-related health inequality—in which, each contribution is the product of the sensitivity of health with respect to that determinant and the degree of income-related inequality in that determinant [28]. Health economists normally put forward the different decomposition methods in the literature to decompose the concentration index. In this study, we employed the decomposition approach from the Wagstaff. In the case that the health sector variables are continuous variables, the decomposition approach based on ordinary least squares regression was commonly adopted [29]. The OLS regression equation between y and x can be written as:

$$y_{i}=\alpha +\sum_{k}\beta_{k}x_{ki}+\varepsilon_{i}$$

where $y_{i}$ is the health sector for *i*, $\beta_{k}$ is the coefficient for kth variable, $x_{k}$ is the kth independent variable, and $\varepsilon_{i}$ is the residual error.

The concentration index for y, can be written as:

$$c=\sum_{k}(\beta_{k}{\bar{x}}_{k}/\mu )c_{k}+GC_{\varepsilon }/\mu$$

where c is the overall concentration index of health sector variable, ${\bar{x}}_{k}$ and $c_{k}$ are the mean and the concentration index of $x_{k}$, and $GC_{\varepsilon }$ is the generalized concentration index for ε. The equation shows that the concentration index is made up of two components: the explained component and residual variation (unexplained) component that was not explained by any of independent variables.

In the health sector, outcome variables are seldom continuous and are often binary. In that case, a marginal effect based on logistic regression could be employed to approximate the decomposition analysis. The logistic regression can be written as follows:

$$\log it(p)=\alpha +\sum_{k}\beta_{k}x_{ki}+\varepsilon_{i}$$

where log*it*(*P*) is a linear function of independent variable, $\beta_{k}$ is the coefficient for kth variable, $x_{k}$ is the kth independent variable, and $\varepsilon_{i}$ is the residual error.

A linear approximation of the non-linear estimations using a marginal effect obtained from logistic regression can be written as follows:

$$y_{i}=\alpha^{m}+\sum_{k}\beta_{k}{}^{m}x_{ki}+u_{i}$$

where $\beta_{k}{}^{m}$ is the marginal effect for kth variable and $u_{i}$ is the error generated by the linear approximation used to obtain the marginal effects. Marginal or partial effects have been analyzed in the analysis of health sector inequalities in non-linear settings. In this case, the concentration index also can be decomposed into two parts, the explained component and the residual variation component

$$c=\sum_{k}(\beta_{k}{}^{m}{\bar{x}}_{k}/\mu )c_{k}+residusial$$

More details on decomposition methods when the dependent variable is binary can be found in previous studies [26,30].

All statistical analyses were performed with SAS version 9.4 and Stata/MP version 14.0.

## 3. Results

Table 1 shows the characteristics of respondents. A total of 12,208 adults, including 5746 (47.07%) men and 6462 (52.93%) women, participated in the study, with a mean (standard deviation, SD) age of 52.59 (15.31) years, and most of them were married. Over 33% of respondents had middle school education. In total, 97.31% of respondents were covered by basic medical insurance and 39.93% lived in urban areas.

**Figure 1:**
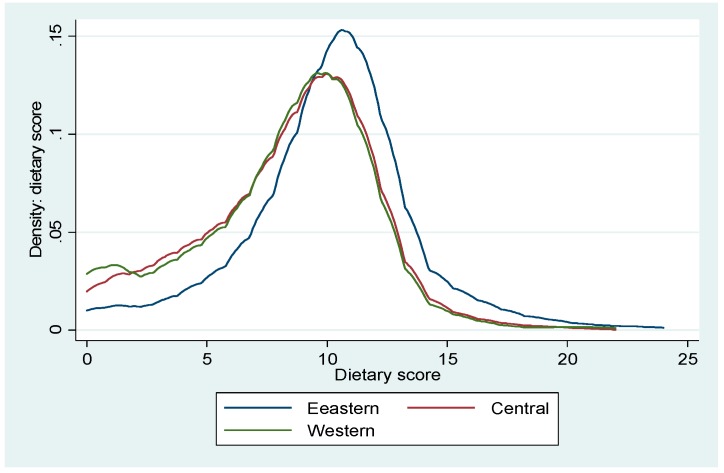
Distribution of dietary scores by geographic region.

Figure 1 shows the distribution of dietary knowledge scores by geographic region. It shows that residents in Eastern China have the highest mean score of dietary knowledge and the thickest right-hand tail on the graph than Central and Western China.

Table 2 shows the means of the dietary knowledge score and dietary guideline awareness by study variables. Univariate analyses showed that there was a statistically significant difference in the dietary knowledge score and dietary guideline awareness among age, income, educational attainment, marital status, working status, residential areas, and geographic region. There was no significant difference in the dietary knowledge score and dietary guideline awareness between women and men. Further post-hoc comparison analysis showed that there was a statistical difference in the dietary knowledge score between Central China and Western China. However, no difference was found in dietary guideline awareness.

Table 3 shows the proportion of respondents who correctly answered dietary knowledge by each question. Urban residents had a higher proportion of correctly answered questions compared to rural residents, except two. “Choosing a diet with a lot of staple foods is not good for one’s health” was the question with lowest score. Only 2077 (42.61%) of urban residents and 3006 (40.99%) of rural residents answered the question correctly.

Table 4 shows the association between determinants and the summary dietary knowledge score/dietary guideline awareness from OLS regression and logistic regression, respectively. Income and educational attainment had a positive association with the dietary knowledge score and dietary guideline awareness. An increase in income and education attainment significantly increased the dietary knowledge score and dietary guideline awareness. The higher the urbanization index, the higher the dietary knowledge score and dietary guideline awareness. Compared to urban residents, rural residents had a lower dietary knowledge score and dietary guideline awareness. Compared to respondents who lived in Eastern China, respondents who lived in Western and Central China had a lower dietary knowledge score and lower dietary guideline awareness.

**Figure 2:**
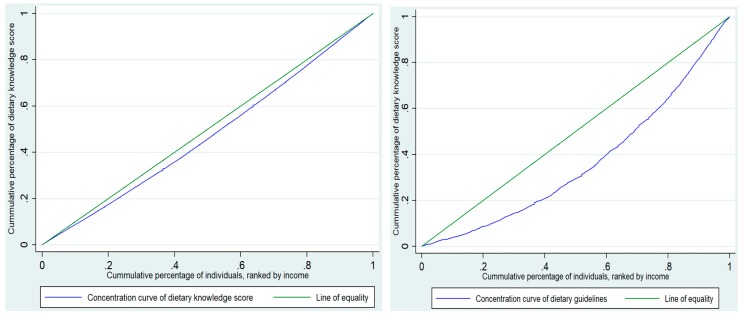
Concentration curves of the dietary knowledge score and dietary guideline awareness, respectively. The green line, running from the lower left corner to the upper right corner, represents the line of equality. The blue line above the equality line is the concentration curve. The farther the concentration curve is below the equality line, the more the health sector variable concentrates among the rich.

Figure 2 shows the concentration curve and the concentration index on the dietary knowledge score and dietary guideline awareness. Both curves lie under the 45-degree line, and the corresponding concentration index was 0.054 ± 0.002 and 0.274 ± 0.008 for the dietary knowledge score and dietary guideline awareness, respectively. That means that there was pro-rich inequality in the dietary knowledge score and dietary guideline awareness. A higher dietary knowledge score and better dietary guideline awareness were more concentrated on the rich.

The decomposition results on the concentration index of the dietary knowledge score and dietary guideline awareness are presented in Table 5. Since the health sector variables of interest were concentrated among the rich, positive contribution means that these variables increase the degree of pro-rich inequality. That is, the rich were more likely to have a higher dietary knowledge score and better dietary guideline awareness. Results showed that the largest and the second largest absolute contributions were from income and educational attainment for the observed inequality of the dietary knowledge score and dietary guideline awareness. These positive contributions were slightly offset by negative contributions from marital status. Quantifying the contributions expressed as the percentage of each independent variable, urbanization index showed 11.868% and 9.135% contribution to the dietary knowledge score and dietary guideline awareness, respectively. Living in Western China contributed 19.46% and 8.16% to the total observed inequality in the dietary knowledge score and dietary guideline awareness, respectively.

## 4. Discussion

Increasing the dietary knowledge of the general population is an important way to improve their dietary habits and lifestyle while reducing incidences of obesity-related non-communicable diseases throughout the whole lifespan [31]. Using the large-scale CHNS data, this study measured the proportion of residents who were aware of dietary guidelines and who correctly answered the dietary knowledge questions. The study found that compared to rural residents, urban residents had a higher proportion of correctly answered dietary knowledge questions in nearly every question. Among all of the questions, “Choosing a diet with a lot of staple foods is not good for one’s health” had the lowest proportion of residents with correct answers for both urban and rural areas. In China’s history, the Chinese suffered from a long period of hard times and lack of food approximately half a century ago. In these hard times, staple food played a prominent role in supplying a large fraction of energy needs to people. Although China’s economic growth has increased rapidly since the open and reform policy, the attitudes and beliefs have not changed much among Chinese people. Chinese people still maintain the traditional habit of eating a lot of staple food, such as rice and noodles. The main function of staple food is compensatory energy. Some staple food can be good for residents in moderation; however, excessive consumption may negatively affect general health. White rice and refined wheat contribute approximately 30% of energy to diets in China. Highly processed carbohydrates, such as white rice, may lead to a quick rise, high peak and fast fall in blood glucose, resulting in high glycemic index and glycemic load values [32]. Too much sugar contained in staple food is a predicator for type 2 diabetes [33]. Currently, most residents, especially in remote rural areas, are still not aware of this. Further actions need to be taken in future public health plans to educate residents to understand the risk of consuming excessive staple food. Despite most people having a clear understanding that physical activities have innumerable physical and mental health benefits, more than half of them do not realize that excessive intense physical activities can be harmful to their health, such as increasing cardiac risks [34]. Slightly larger gaps were observed between urban and rural residents in term of answering questions related to sugar consumption and fat consumption. Compared to other questions, these two questions showed a significant difference, with urban residents providing more correct answers than rural residents. Recent studies found that various fats influence health differently, e.g., unsaturated fats are considered to play a number of beneficial roles in health, while other fats, such as trans fats, are considered to do the opposite. There is still a lot that can be done to improve people knowledge regarding this topic, especially in rural areas.

Some key factors were identified as the determinants of the dietary knowledge score and dietary guideline awareness in this study. As expected, educational attainment was associated with the dietary knowledge score and dietary guideline awareness. People with a higher level of education are more likely to be health literate, take the initiative to seek dietary information for health considerations, achieve a better understanding of nutritional information from mass media, and achieve better informed judgment ability to distinguish between correct and incorrect commercial promotions. In this study, as a continuous variable, the urbanization index was used to explore the association between the dietary knowledge score and dietary guideline awareness. The study found that there were positive relationships between the dietary knowledge score/dietary guideline awareness and the urbanization index. A feasible reason for this is that residents who live in communities with a higher urbanization level are likely to have more nutritional education from school, and more opportunity to access to health knowledge correctly. Our unpublished data show that 38.65% of respondents who lived in communities with a higher urbanization index proactively looked for nutrition information, while this figure was only 15.55% in communities with a lower urbanization index. Less developed communities commonly use TV and radio as sources for nutritional knowledge. However, mass media is full of fast food advertisements, which usually has lower nutritional benefits. Combined with relatively poor analysis skills and judgment ability, residents who lived in lower urbanization communities are prone to lower dietary knowledge. Geographic region is another factor associated with dietary knowledge. Due to geographical conditions, residents in Central and Western China may lack the resources for better dietary knowledge, limiting the possibility to acquire nutrition knowledge from media, and less opportunity to visit dietitians. Regarding marital status, unlike previous studies, individuals who were married had a higher score on dietary knowledge and better dietary guideline awareness. That may be due to the fact that people who are married are most likely to share their nutrition information with each other [17]. This study confirmed the findings from previous studies that women had a higher dietary knowledge than men. Men are less interested in nutritional knowledge than women. Previous studies showed that more than 25% of men were unwilling to increase their knowledge about food and nutrition [35].

Previous studies on nutritional knowledge found that nutritional knowledge was not distributed equally among residents with different incomes [4]. The study also observed a dietary knowledge difference among income groups, and further measured the magnitude of this inequality. This study found that people with a higher income were more likely to have higher dietary knowledge. The implementation of dietary education may motivate residents to change their dietary behaviors and provide them with the knowledge and skills to make healthy food choices in the context of their lifestyles and economic resources. This study recommends future dietary education and promotion programs to provide different strategies for individuals with different educational levels. Furthermore, these programs should be made more available to disadvantaged people. The contents of education should be adapted to local dietary habits. This would make it easier for local residents to understand and adopt these practices. This study further explored the source of this observed inequality. As expected, income and educational attainment showed the largest proportion of contribution on pro-rich inequality in dietary knowledge sore and dietary guideline awareness. However, the contribution of sub-educational attainment groups was heterogeneous. Elementary and middle school education showed a negative contribution to this pro-rich inequality, whereas high school and university education showed a positive contribution to this kind of inequality. That is because the poor are more likely to have had elementary or middle school attainment, whereas the rich are more likely to have had high school and university education. The study showed that geographic region contributed a large proportion to observed inequality. The possible explanation for this is as follows. Compared to residents living in Eastern China, residents living in Central and Western China had a lower score of dietary knowledge and a lower likelihood of awareness of dietary guidelines. This may be due to the fact that Central and Western China are relatively less prosperous compared to the Eastern China. Increasing the economic investments in Central and Western China, which may lead to a reduced gap of income, could be a feasible way to decrease this kind of inequality.

This study has some limitations. Firstly, due to the cross-sectional study design, causal inference between dietary knowledge and independent variables cannot be established. Secondly, since all of the data were self-reported, respondents may not fully remember past events or experiences accurately or omit details. Therefore, recall bias is unavoidable. To limit recall bias, different recall periods were used in the CHNS. Thirdly, the determinants of the dietary knowledge score and dietary guideline awareness considered in the study were from the pre-specified questions in the survey. Other unobserved confounders were not considered in the regression model.

## 5. Conclusions

Using a national-scale survey, this study measured the dietary knowledge of Chinese adults. The study found that the proportion of respondents who correctly answered dietary knowledge questions was significantly low for some questions. Income, educational attainment, the urbanization index, and geographic region were the main determinants for both the dietary knowledge score and dietary guideline awareness. There was pro-rich inequality in the dietary knowledge score and dietary guideline awareness. Income and educational attainment appeared to be the first and second largest proportion of contrition on this pro-rich inequality. In addition, the urbanization index and geographic region also showed large contributions to the observed inequality. This study recommends that future dietary education incorporates different strategies for individuals with different educational levels, with more focus on disadvantaged people. It would be beneficial to consider local dietary habits in developing education materials. This approach could greatly assist local residents in understanding and adopting something they are familiar with to improve their knowledge and to embrace healthy diets and lifestyles.

## Figures and Tables

**Table 1:** Characteristics of respondents.

Characteristics	All (N = 12,208)
Age, years	
Mean (SD)	52.59 ± 15.31
Gender	
Men, n (%)	5746 (47.07)
Women, n (%)	6462 (52.93)
Missing	
Income, RMB yuan	
Mean (SD)	24942.94 ± 39654.58
Education	
Illiterate, n (%)	871 (7.13)
Elementary, n (%)	2821 (23.11)
Middle school, n (%)	4069 (33.33)
High school, n (%)	2937 (24.06)
University, n (%)	1510 (12.37)
Marital status	
Unmarried, n (%)	648 (5.31)
Married, n (%)	10,539 (86.33)
Others, n (%)	1021 (8.36)
Basic medical insurance	
No, n (%)	328 (2.69)
Yes, n (%)	11,880 (97.31)
Working status	
No, n (%)	6445 (52.79)
Yes, n (%)	5763 (47.21)
Birthplace	
North China, n (%)	965 (7.90)
Northeast, n (%)	1714 (14.04)
East China, n (%)	3244 (26.57)
Central China, n (%)	3063 (25.09)
South China, n (%)	1191 (9.76)
Western China, n (%)	2031 (16.64)
Urbanization index	
Mean (SD)	73.98 ± 17.07
Residential areas	
Urban, n (%)	4875 (39.93)
Rural, n (%)	7333 (60.07)
Geographic region	
Eastern China, n (%)	5942 (48.67)
Central China, n (%)	3074 (25.18)
Western China, n (%)	3192 (26.15)

**Table 2:** Univariate analysis on the dietary knowledge score and dietary guideline awareness.

	Dietary Knowledge Score				Dietary Guideline Awareness			
	Mean	S.D.	F/t	*p*	n	%	χ^2^	*p*
Age			25.52	<0.001			94.60	<0.001
18−44	9.41	3.72			1226	33.23		
45−59	9.17	3.55			1134	27.24		
≥60	8.82	4.01			1025	23.53		
Post-hoc test	“18−44” > “45−59” > “≥60”				“18−44” > “45−59” > “≥60”			
Gender			0.77	0.44			0.81	0.37
Men	9.14	3.77			1571	27.34		
Women	9.09	3.78			1814	28.07		
Income			167.76	<0.001			1072.66	<0.001
Poorest	7.95	3.89			291	11.92		
Poorer	8.36	3.72			412	16.89		
Middle	9.19	3.52			643	26.38		
Richer	9.84	3.44			830	33.92		
Richest	10.23	3.80			1209	49.47		
Post-hoc test	Richest > Richer > Middle > Poorer > Poorest				Richest > Richer > Middle > Poorer > Poorest			
Educational attainment			187.41	<0.001			1776.81	<0.001
Illiterate	7.20	3.92			34	3.90		
Elementary	8.24	3.70			328	11.63		
Middle school	9.12	3.51			882	21.68		
High school	9.75	3.68			1232	41.95		
University	10.64	3.81			909	60.20		
Post-hoc test	University > High school > Middle school > Elementary > Illiteracy				University > High school > Middle school > Elementary > Illiteracy			
Marital status			20.58	<0.001			50.70	<0.001
Unmarried	9.23	3.91			213	32.87		
Married	9.18	3.74			2980	28.28		
Others	8.39	4.01			192	18.81		
Post-hoc test	Unmarried, Married > Others				Unmarried > Married > Others			
Basic medical insurance			−4.48	<0.001			0.55	0.46
No	8.20	4.27			85	25.91		
Yes	9.14	3.76			3300	27.78		
Working status			−9.16	<0.001			109.22	<0.001
No	8.82	3.94			1529	23.72		
Yes	9.45	3.55			1856	32.21		
Residential areas			14.87	<0.001			544.53	<0.001
Urban	9.73	3.83			1917	39.32		
Rural	8.71	3.69			1468	20.02		
Geographic region			375.75	<0.001				
Eastern China	10.05	3.60			2232	37.56	559.25	<0.001
Central China	8.35	3.64			577	18.77		
Western China	8.12	3.80			576	18.05		
Post-hoc test	Eastern China > Central China> Western China				Eastern China > Central China, Western China			

Note: The post-hoc Tukey test was conducted to determine which groups differ from each other. S.D., Standard deviation.

**Table 3:** The number and proportion of respondents who correctly answered questions on dietary knowledge.

Questions on Dietary Knowledge	Urban		Rural		χ^2^	*p*
	n	%	n	%		
Choosing a diet with a lot of fresh fruits and vegetables is good for one’s health	3903	80.06	5424	73.97	60.33	<0.001
Eating a lot of sugar is good for one’s health	3823	78.42	5150	70.23	100.85	<0.001
Eating a variety of foods is good for one’s health	3858	79.14	5413	73.82	45.39	<0.001
Choosing a diet high in fat is good for one’s health	3806	78.07	5081	69.29	114.05	<0.001
Choosing a diet with a lot of staple foods is not good for one’s health	2077	42.61	3006	40.99	3.13	0.077
Consuming a lot of animal products daily is good for one’s health	3211	65.87	4467	60.92	30.75	<0.001
Reducing the amount of fatty meat and animal fat in the diet is good for one’s health	3560	73.03	4997	68.14	33.28	<0.001
Consuming milk and dairy products is good for one’s health	4098	84.06	5990	81.69	11.52	0.000
Consuming beans and bean products is good for one’s health	4064	83.36	6153	83.91	0.64	0.425
Physical activities are good for one’s health	3866	79.30	5667	77.28	7.00	0.008
Sweaty sports or other intense physical activities are not good for one’s health	2324	47.67	3179	43.35	22.07	<0.001
The heavier one’s body is, the healthier he or she is	3908	80.16	5477	74.69	49.37	<0.001

**Table 4:** Adjusted association between the dietary knowledge score and dietary guideline awareness.

Variables	Dietary Knowledge Score			Dietary Guideline Awareness		
	Estimate	Std. Err.	*p*	Estimate	Std. Err.	*p*
Age (years)	−0.007	0.003	0.015	0.000	0.002	0.950
Gender						
Men (ref)						
Women	0.149	0.068	0.028	0.252	0.048	<0.001
Income						
Poorest (ref)						
Poorer	0.065	0.103	0.524	0.082	0.088	0.351
Middle	0.515	0.106	<0.001	0.379	0.085	<0.001
Richer	0.805	0.110	<0.001	0.463	0.085	<0.001
Richest	0.768	0.118	<0.001	0.798	0.088	<0.001
Education						
Illiterate (ref)						
Elementary	0.841	0.141	<0.001	1.059	0.181	<0.001
Middle school	1.342	0.146	<0.001	1.641	0.179	<0.001
High school	1.479	0.156	<0.001	2.340	0.181	<0.001
University	1.871	0.183	<0.001	2.821	0.191	<0.001
Marital status						
Unmarried (ref)						
Married	0.521	0.156	0.001	0.392	0.107	<0.001
Others	0.359	0.202	0.076	0.258	0.147	0.079
Basic medical insurance						
No (ref)						
Yes	1.207	0.200	<0.001	0.292	0.143	0.041
Working status						
No (ref)						
Yes	0.191	0.075	0.011	0.111	0.055	0.041
Birthplace						
North China (ref)						
Northeast	−0.602	0.147	<0.001	−0.380	0.093	<0.001
East China	−0.420	0.132	0.001	−0.187	0.081	0.021
Central China	−0.915	0.422	0.030	0.026	0.275	0.926
South China	0.306	0.461	0.506	0.136	0.301	0.652
Western China	−0.481	0.453	0.288	−0.206	0.293	0.482
Urbanization index	0.015	0.002	<0.001	0.010	0.002	<0.001
Residential areas						
Urban (ref)						
Rural	−0.353	0.074	<0.001	−0.370	0.051	<0.001
Geographic region						
Eastern China(ref)						
Central China	−0.804	0.410	0.050	−0.852	0.269	0.002
Western China	−1.648	0.443	<0.001	−0.667	0.287	0.020

Note: Std. Err., Standard error.

**Table 5:** Decomposition analysis of the concentration indexes on the dietary knowledge score and dietary guideline awareness.

Variables	Dietary Knowledge Score				Dietary Guidelines			
	Elasticity	C_K_	Absolute Contribution to C	Percentage Contribution to C	Elasticity	C_K_	Absolute Contribution to C	Percentage Contribution to C
Age (years)	−0.041	−0.003	0.000	0.200	0.004	−0.003	0.000	−0.004
Gender								
Men (ref)								
Women	0.025	−0.003	0.000	−0.148	0.243	−0.003	−0.001	−0.283
Income								
Poorest (ref)								
Poorer	0.001	−0.401	−0.001	−1.065	0.010	−0.401	−0.004	−1.528
Middle	0.011	−0.001	0.000	−0.020	0.051	−0.001	0.000	−0.018
Richer	0.018	0.399	0.007	13.123	0.063	0.399	0.025	9.187
Richest	0.017	0.800	0.013	24.989	0.113	0.800	0.091	33.047
Education								
Illiterate (ref)								
Elementary	0.021	−0.210	−0.004	−8.288	0.176	−0.210	−0.037	−13.477
Middle school	0.049	−0.070	−0.003	−6.361	0.386	−0.070	−0.027	−9.870
High school	0.039	0.172	0.007	12.413	0.426	0.172	0.073	26.696
University	0.025	0.432	0.011	20.307	0.270	0.432	0.117	42.546
Marital status								
Unmarried (ref)								
Married	0.049	0.003	0.000	0.318	0.196	0.003	0.001	0.250
Others	0.003	−0.099	0.000	−0.604	0.014	−0.099	−0.001	−0.520
Basic medical insurance								
No (ref)								
Yes	0.129	0.001	0.000	0.306	0.166	0.001	0.000	0.078
Working status								
No(ref)								
Yes	0.010	0.082	0.001	1.511	0.033	0.082	0.003	0.998
Birthplace								
North China (ref)								
Northeast	−0.009	0.053	0.000	−0.905	−0.031	0.053	−0.002	−0.598
East China	−0.012	0.219	−0.003	−4.972	−0.031	0.219	−0.007	−2.452
Central China	−0.025	−0.143	0.004	6.684	0.004	−0.143	−0.001	−0.214
South China	0.003	−0.318	−0.001	−1.933	0.009	−0.318	−0.003	−1.000
Western China	−0.009	−0.154	0.001	2.501	−0.021	−0.154	0.003	1.169
Urbanization index	0.120	0.053	0.006	11.868	0.469	0.053	0.025	9.135
Residential areas								
Urban (ref)								
Rural	−0.062	−0.048	0.003	5.524	−0.374	−0.048	0.018	6.560
Geographic region								
Eastern China (ref)								
Central China	−0.022	−0.144	0.003	5.935	−0.120	−0.144	0.017	6.305
Western China	−0.047	−0.222	0.011	19.461	−0.101	−0.222	0.022	8.161

Note: C, the concentration index; C_K_, the concentration index for the k_th_ independent variable; ref, reference group.
